# Gonorrhea as a Social Problem

**Published:** 1902-02

**Authors:** 


### GONORRHEA AS A SOCIAL PROBLEM.

THIS is a question which has engaged the attention of the profession for a long time, but it was not, however, until Moggerath made his monumental announcement of the role played by the disease in the production of pelvic disorders in women that the question took on a new and more vital interest. The literature on the subject is vast, and out of it all it may be gleaned that gonorrhea retains its virulence for a long period, and that it is responsible for a great many of the diseases of the procreative apparatus of women. It would seem that a dissent from the prevailing opinion on this subject would be of little avail and be regarded as an irrational attempt to controvert a proven and established fact; yet it must be remembered that persistence in certain investigations is apt to warp the mind of the investigator and to give rise to extravagance in statement. There seems but little question that gonorrhea is a serious disease, one of unknown duration, and that its ravages, after being installed in the genital passages of women, are extensive, but there is also little question, to any one with experience, that not all women are infected who are exposed to an old gonorrhea in the man, or that after infection the disease is always severe or mutilating.

It is a matter of common observation to see a man with a chronic urethral discharge in possession of a healthy and fruitful wife, and the intimacy of married life is a searching test of the infectiousness of a disease in either of the couple. They are subjected to all phases in the state of the discharge, and if the discharge was virulent infection would be inevitable. The argument that habituation of the tissues confers immunity or conditions the course of the disease does not fully explain this observation. Infection, mild or severe, must have occurred to confer immunity, while the contention in this instance is that no infection whatever has taken place. How often this escape from infection occurs in the history of gonorrheal exposure, and what ratio it bears to actual infection is, of course, a matter of conjecture; but, taking into consideration the universal prevalence of the disease among men and the greater frequence of sexual relations during the inveterate stage of the disease, it seems likely that more women escape infection than are infected. As gonorrhea in women is alleged to be the prime cause of sterility, and if, as Moggerath asserted, probably correctly, of 1,000 women 800 were married to ex-gonorrheics, the world would be depopulated were contamination to occur regularly.

This contradictory attitude of well-known facts and observations does not militate against much of valuable truth that gynecologists have brought forward, but it shows that enthusiasm in endeavoring to establish a principle may carry beyond the legitimate limits of fact.

---

AS THE new Marine Hospital on Ellis Island, New York, was nearing completion, it was discovered that no provision had been made for lighting the building. The contractors showed that the specifications contained no mention of artificial illumination, and so exonerated themselves. Parts of the building will have to be removed in order to repair the defect.

